# The impact of sanction and healthcare system reform on the healthcare performance and pharmaceutical market in Iran; 2001-2016

**DOI:** 10.1186/s40545-020-00245-z

**Published:** 2020-08-27

**Authors:** Saman Zartab, Nasrin Nassiri Koopaei, Hadi Abbasian, Mansur Nassiri Koopaei, Nasser Nassiri Koopaei

**Affiliations:** 1 Department of Pharmacoeconomics and Pharmaceutical Administration, Faculty of Pharmacy, Tehran University of Medical Sciences, Tehran, Iran; 2 Department of Medicinal Chemistry, Faculty of Pharmacy, Pharmaceutical Sciences Branch, Islamic Azad University, Tehran, Iran; 3 Food and Drug Department of Shahid Beheshti University of Medical Sciences, Tehran, Iran; 4 Department of Medicinal Chemistry, Faculty of Pharmacy Pharmaceutical Sciences Research Center, Tehran University of Medical Sciences, Tehran, Iran; 5 Department of Pharmaceutical Engineering, School of Chemical Engineering, University of Tehran, 16th Azar St., Enghelab Sq, Tehran, 1981165134 Iran

**Keywords:** Healthcare system reform, Pharmaceutical market trend analysis, Financial indicators, Performance assessment, Drug consumption

## Abstract

**Background:**

Iranian government has introduced multiple healthcare system reforms during the last 30 years aiming at improving accessibility and affordability of care. Pharmaceutical products are one of the major sources of financial burden on the healthcare system. The healthcare system and pharmaceutical sector have been balanced out by the partially counteracting effects of the HSEP (Health sector evolution plan) and the imposed sanctions.

**Methods:**

This research investigates the healthcare system performance as well as the pharmaceutical market trend mostly based on the financial criteria from 2001. The correlation between the two change patterns was studied to understand the underlying driving market forces.

**Results:**

During 2001 to 2013, total health expenditure has grown 25.6% in average. THE (Total health expenditure) share of the GDP remains between 6-7%, while the out of pocket payment has dropped to 37% in 2015 from 57% in 2001, and most health services been directed to the inpatient facilities. Iranian pharmaceutical market has grown rapidly in recent years and grew 28.38% per year and drug consumption per capita reached 34.43$ from 2.28$. However, the import drove most of the market expansion. Noteworthy, the share of pharmaceuticals from THE has also increased.

**Conclusions:**

It is concluded that the sanctions and HSEP have enforced partially counteracting forces on the pharmaceutical market to maintain its consistent growing trend.

## Background

Iranian government has introduced multiple healthcare system reforms during the last 30 years with the aim of improving accessibility and affordability of care [1, 2]. Historically, these efforts included enforcement of the national health network, family physician program, integration of healthcare services and medical education, hospital autonomy plan, and the latest called health sector evolution plan (HSEP) designed and implemented by the Ministry of Health and Medical Education (MoH) in 2014 with a stepwise implementation. HSEP tried to transform the healthcare system in public hospitals to mitigate the dramatic healthcare cost surge [1, 3, 4]. The plan was sold with a positive public opinion to reduce the burden of healthcare expenditure on the patients by providing the health insurance coverage to the uninsured population and decrease in patient copayment and deductible, revolutionize the hospital organization, provide quality care, redistribution of providers, and making the inpatient care accessible to all, especially orphan diseases. As of the introduction, the patients and providers reacted positively because of the cost reduction in demand side and the increase in the revenues on the supply side [1, 5, 6]. As expected, the popular plan faced critical hurdles including drastic government spending, systemic reimbursement failure, and financial resources. The HSEP did not also cover the prevention and primary healthcare, outpatient services, and the private sector [1, 7, 8]. The financial burden reduction is primarily financed by an increase in government spending, the health-related subsidies and value-added tax on goods [9].

Pharmaceuticals is one of the major sources of financial burden on the healthcare system [10–12]. The healthcare system and pharmaceutical sector have been facing the partially counteracting effects of the HSEP and the imposed sanctions [13, 14]. Iranian pharmaceutical market has grown rapidly in recent years. During 1997-2010, the population has grown 1.53% annually, but, the pharmaceutical market grew 28.38% per year and drug consumption per capita reached 34.43$ from 2.28$. Although, the pharmaceutical expenditure is a concern for many countries and policymakers, but, in certain situations, it turned into a national crisis attributed mostly to the sanctions [15–17]. However, the pharmaceutical import accounted for the most part of the market expansion which indicates that the growth could have been because of other booming sectors of the economy, especially, petrochemical industry and oil revenues which lacked sustainability. In this period, domestic production grew 1018% while importation grew 4373%. Noteworthy, the biopharmaceutical sector did not attract remarkable investment [18, 19]. The current study tries to elaborate on the effects of the two aforementioned factors including HSEP and sanctions on the Iranian pharmaceutical market and the evolution of the industry and the market during the market instability period. This research investigates the healthcare system performance as well as the pharmaceutical market trend mostly based on the financial criteria from 2001. Then, we try to find the correlation between the two change patterns to understand the underlying driving forces in the market.

## Methods

This research is a descriptive cross-sectional study investigating the Iranian healthcare sector economy and pharmaceutical market in 2001 to 2016, wherever data could be obtained as explained further in the results section. The healthcare expenditure data was gathered from the Statistical Center of Iran or the Iranian MoH databases [11, 20–22]. The drug consumption data was extracted from the Iranian pharmaceutical statistical datasheet published by the Iranian Food and Drug Administration (IFDA) through the data received from the drug distribution companies published annually or biennially. It is assumed that the sales data from the distribution companies as the sole supply source of drugs to the pharmacies under the law is a surrogate for drug consumption. Population data was extracted from the Statistical Center of Iran database and currency exchange rate and the Gross Domestic Product (GDP) data extracted from the Central Bank of Iran website. All the statistical analyses were carried out using MS Excel 2013.

## Results

**Fig. 1:**
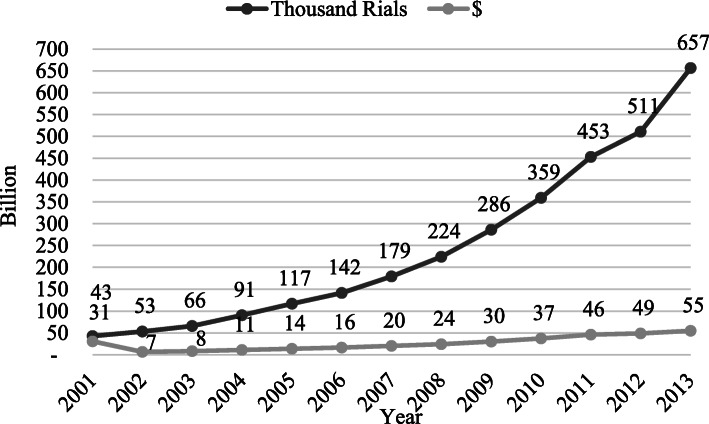
THE trend in Iran; 2001-2013

During 2001 to 2013, total health expenditure has grown 25.6% in average. Because of the inflationary national economy, the total health expenditure (THE) has grown 5% annually which has decreased steadily and reached −7% in 2013 from 17% in 2004. As Fig. 1 shows, the THE has decreased for the first time in 2012 and 2013 based on a fixed-weight price index. Although in these 2 years, the GDP and THE assuming fixed-weight price index have both decreased, but, the THE has decreased more rapidly than the GDP; therefore, the healthcare cost share from the GDP dwindled. National Health Accounts (NHA) data show that the direct health expenditure constituted 95% of THE on average and indirect health expenditure just accounted for 5% of THE (2001-2013).

**Table 1:** Major players in Iranian pharmaceutical market (2015)

Rank	Holding group	Sales value ($)	Market share (%)	Cumulative share (%)	Ownership
**1**	TPICO	657,753,710	17.5	0.17	Public
**2**	Cobel Darou	552,089,105	14.7	0.32	Private
**3**	Shafa Darou	293,516,423	7.8	0.40	Public
**4**	Behphar Holding	274,924,535	7.3	0.47	Private
**5**	Alborz Investment	263,503,586	7.0	0.54	Public
**6**	Tehran Chemie	197,998,341	5.3	0.60	Private

**Fig. 2:**
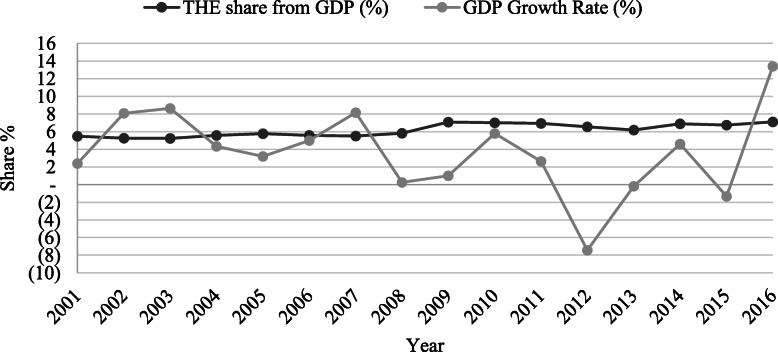
THE share of the GDP and GDP growth rate; 2001-2016

However, as Fig. 2 shows, the THE share from GDP has been relatively stable from 2009 to 2016, although the GDP growth rate has been fluctuating significantly.

**Fig. 3:**
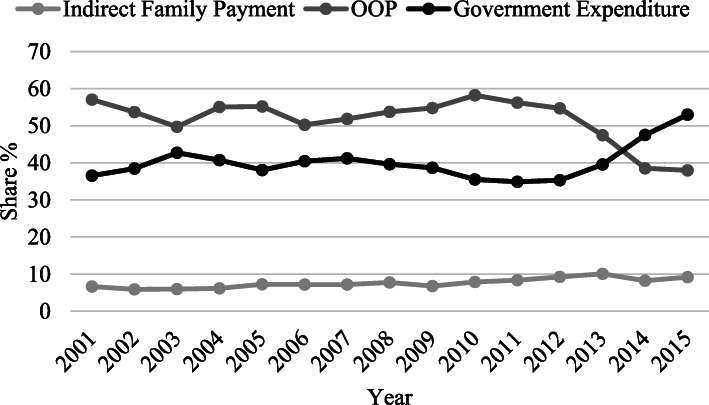
Share of healthcare expenditure coverage sources; 2001-2015

Financing healthcare cost has long been the challenging factor in healthcare accessibility and affordability. The three most important sources for healthcare cost reimbursement are household payment, governmental reimbursement, employer payment, and other sources that have accounted for 58.15%, 26.81%, 9.67%, and 5.37% in 2001-2013 on average, respectively. As Fig. 3 shows, household payment and governmental reimbursement have worked mutually and reciprocally to cover the costs. Figure 3 shows the share of healthcare cost reimbursement from THE broken into direct household payment as out-of-pocket payment (OOP), government health expenditure (GHE), and employer payment and indirect household payment as premiums. The OOP has reached 37% in 2015 from 57% in 2001 which shows a 20% decrease. However, the increased share of the government and employers in the THE compromises the sustainability of the healthcare system reform financing that remains a challenge for the authorities, because the government share is mostly backed by the oil revenues.

**Fig. 4:**
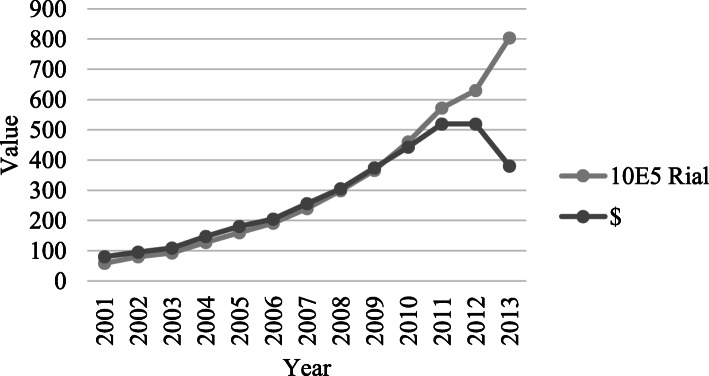
THE per capita trend; 2001-2013

THE per capita has grown to 380$ in 2013 from 79$ in 2001 (Fig. 4). High inflation rate and national currency devaluation which boomed in 2010, caused the THE per capita in dollar and rial diverge, and in 2012 and 2013 contradict significantly.

Table 1 breaks down the pharmaceutical market into its major sectors. As is evident, market concentration is low, and governmental or public companies dominate the national market. But, during the recent years, private companies are gaining more market share.

Table 2 shows the top ten Iranian pharmaceutical companies in 2016. As the data suggests, most of the companies are public in the production sector; however, the private companies are gaining remarkable market share specifically by launching high-tech products and biological drugs. The market shares also denote lack of market concentration in comparison to the import sector.

**Table 2:** Top ten domestic production pharmaceutical companies in Iran; 2016

Rank	Company	Sales ($)	Market share (%)	Cumulative share (%)	Ownership
**1**	Dr Abidi	173,194,903	4.59	4.59	Private
**2**	Darou Pakhsh Pharma	169,064,903	4.48	9.07	Public
**3**	Actover	139,703,765	3.70	12.78	Private
**4**	Exir Pharma	133,209,646	3.53	16.31	Public
**5**	Dana	131,028,469	3.47	19.79	Public
**6**	Zahravi	124,236,571	3.29	23.08	Public
**7**	Alborz Darou	117,867,586	3.13	26.20	Public
**8**	Jaber Ebne Hayyan	114,024,975	3.02	29.23	Public
**9**	Cinagen	113,924,849	3.02	32.25	Private
**10**	Tehran Chemie	102,912,559	2.73	34.98	Private

Moreover, the import sector is almost utterly dominated by private companies with high market concentration indices (Table 3). However, the importers are investing heavily on the joint manufacturing facilities with multinational partners to produce under-license or through contract manufacturing which is partially because of policies enforced by the Iranian FDA to limit finished product import.

**Table 3:** Top ten importer companies in Iran; 2016

Rank	Company	Sales ($)	Market share (%)	Cumulative share (%)	Ownership
**1**	Cobel	294,940,054	18.83	18.83	Private
**2**	Behestan Darou	285,046,969	18.20	37.03	Private
**3**	Novonordisk Pars	127,368,336	8.13	45.16	Private
**4**	Shafayab Gostar	120,107,326	7.67	52.83	Private
**5**	Rougine Darou	62,441,690	3.99	56.82	Private
**6**	Avin Darou	50,215,281	3.21	60.03	Private
**7**	Ahran Tejarat	48,767,569	3.11	63.14	Private
**8**	Daryan Salamat	42,833,940	2.73	65.87	Private
**9**	Darman Ara	40,247,166	2.57	68.44	Private
**10**	Kar-O-Andisheh	36,212,998	2.31	70.76	Private

**Fig. 5:**
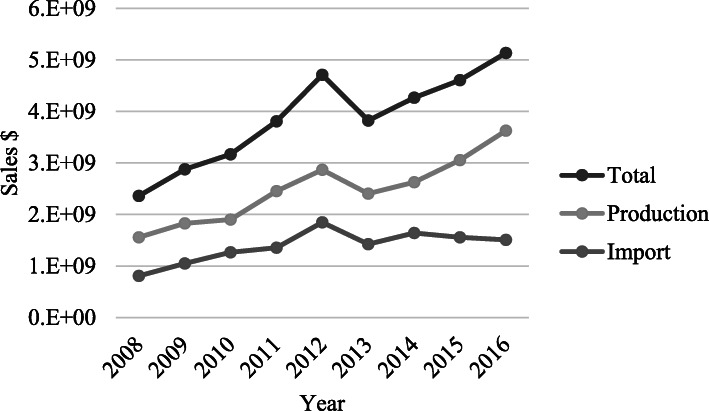
Drug consumption trend in 2008-2016

Market analysis shows that the Iranian pharmaceutical market grew overwhelmingly. The population of the country rose to 79.9 million from 72.2 million. The market size grew to 4.608$ billion in 2016 from 2.358$ billion in 2008 (Fig. 5).

**Fig. 6:**
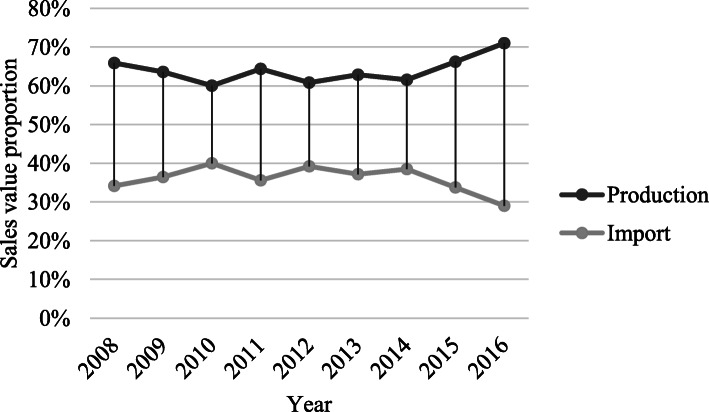
Import and domestic production ratio to the total market size trend in 2008-2016.

The overall and CAGR^1^ of the importation was 93.2% and 9.87%, despite the tight import and pricing policies and support for the domestic production put in place by the Iranian FDA. The import sales values were 0.8$ billion that grew to 1.5$ billion in 2016, but, import to total consumption ratio was relatively stable and fluctuating in 33.7% to 39.2 range (Fig. 6).

**Fig. 7:**
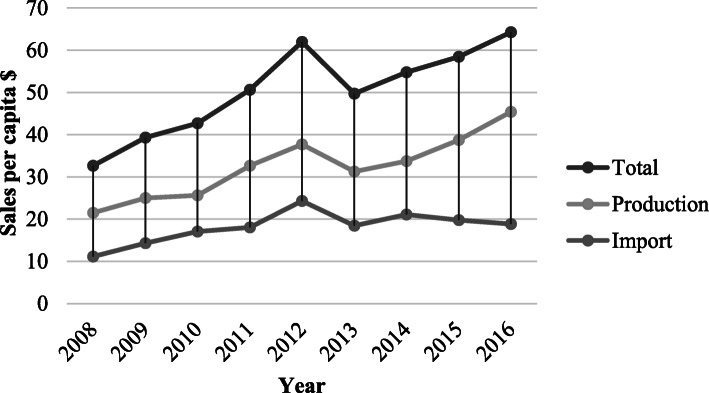
Drug consumption per capita

Another indicator for the market evolution is the import and local production ratio to total consumption per capita. During the past 8 years (2008-2016), drug consumption per capita has reached 58$ from 33$. However, it has been between 51$ to 62$. It seems that the drug consumption per capita growth rate has slowed down after the enforcement of the healthcare system reform and the immediate effects of the imposed crippling sanctions (Fig. 7).

In the past 8 years, local product consumption per capita was between 22$ and 39$, but, the value fluctuated between 31$ to 39$ in the recent 5 years that is relatively stable. This may imply that the enforcement of the healthcare system reform and the short term effects of the sanctions have neutralized their counteracting effects which can be phrased as the channeling and blocking of monetary resources into the healthcare system, respectively. At the same time, imported drug consumption per capita was between 11$ to 24$ while it was between 18$ and 24$ in recent 5 years. This effect is also in line with that of the local products. Because of the Iranian FDA policies to contain the costs and its support of the local production, the funds were mostly funneled into the local manufacturing industries that are predominantly public entities, and therefore, the imported product consumption was tightly harnessed under governmental control. However, this is an approach backed up by international health bodies including the World Bank to improve access to medicines for developing countries [23].

Then, we calculated the concentration ratio for the Iranian pharmaceutical market in 2016 using the following formula:

$$ {\mathrm{CR}}_{\mathrm{m}}={S}_4+{S}_3+{S}_2+{S}_1 $$

*S*_n_ is the market share of company N

If CRm < 40, then perfect competition market

If CRm > 40, then monopolistic market

$$ {\mathrm{CR}}_{\mathrm{m}}=\left(\mathrm{Exir}\right)3.86+\left(\mathrm{Actover}\right)4.04+\left(\mathrm{Abidi}\ \right)4.34+\left(\mathrm{Daroupakhsh}\ \right)4.92=17.16 $$

which shows the market is satisfactorily competitive.

### Drug consumption based on the therapeutic categories

**Fig. 8:**
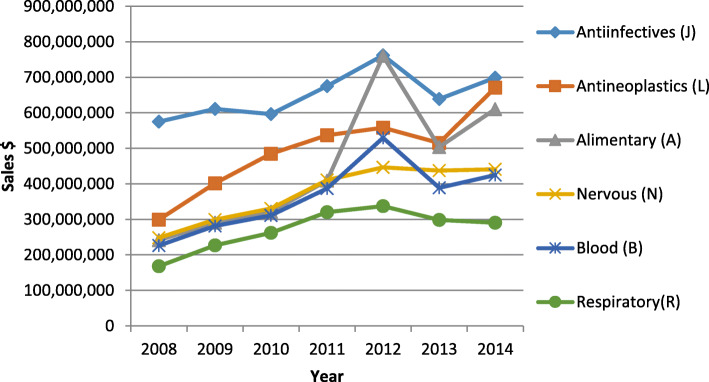
Drug consumption based on the ATC therapeutic categories

Antibiotics for systemic use were the top group in terms of drug consumption. Antineoplastic and immunomodulatory drugs experienced the highest growth rate on which the sanctions and healthcare system reform had the smallest effect. However, the alimentary tract and metabolism drugs had the highest short term growth while they were highly influenced by the sanction and reform. Nonetheless, CNS drugs faced a plateau and respiratory drugs experienced a market loss (Fig. 8).

## Discussion

The current research studies the effects of the healthcare system reform and sanctions on the Iranian pharmaceutical market. In the study period, accessibility and to some extent affordability of health care services and health insurance coverage has significantly increased [24]. Before the healthcare reform act was enforced, the insurance reimbursement did not cover the health care costs for most of the people, and consequently, out of pocket payment was increasing dramatically [25, 26]. As of the reform act going into effect, the Health Insurance Organization of Iran, Social Security Organization, and Armed Forces Health Services Organization were directed to insurance coverage expansion. The expanded insurance coverage tackled the sanctions and market instability and did not let the supply chain fail [27]. Moreover, adequate investment in production of the biotechnology and other high tech product lines prevented the market explosion and failure, in spite of the launch of new products in these categories that helped maintain the local production to import ratio during the investigated period. The market size was 2.35$ billion in 2008 and increased to 4.71$ billion in 2012 right before the sanctions took effect. But, the market dropped to 3.82$ billion pursuant to the imposition of the sanctions. The healthcare system reform pushed the market back to the rail, gaining 4.6$ billion value near that of the before the sanctions which was mostly driven by the local production.

The healthcare system reform did not change the corporate ownership structure in the pharmaceutical industry which still remains more than 70% governmental or public [28]. The annual growth rate and CAGR of the market were 48.67% and 21.93% before and 20.49% and 9.77% after the sanctions, respectively. The annual growth rate and CAGR of the importation were 45.79% and 20.75% before and 9.54% and 4.66% after the sanctions, respectively. Currently, 20% of all the healthcare costs is allocated to the pharmaceuticals. Antibiotics for systemic use, antineoplastics and immunomodulatory drugs, alimentary tract and metabolism drugs, nervous system drugs, and blood products constitute the main ATC groups with the highest sales. However, inappropriate administration of antibiotics has raised concerns about the increased antibiotic resistance [29, 30]. Among these ATC groups, alimentary tract and metabolism drugs experienced the most severe fluctuation in the sanction era which was because of the fact that they are not highly critical and had relatively low prices. Strict governmental control on the drug pricing and importation restrictions has led to a relatively slow pharmaceutical market growth while the healthcare market has been growing during the reform. On the other hand, as new private companies enter the market the share of the large pharmaceutical holding groups has been shrinking and the market grows more competitive.

Healthcare system reform should target tangible and critical set points in the system with well-informed and sustainable measures to address accessibility, affordability, and equity in the health outcomes. The pharmaceutical market and industry are inherently subject to changes in its overarching system; the holistic national economy as an industry with profitability obligation to prosper and, on the other hand, the healthcare system as the overall market in which they have to do business [31, 32]. The results show that the market is very unstable during the sanction era when shocking waves of drug shortage not only on the market troubled the community but also the politicians. The HSEP pushed the prescription practice toward generic products and supply through governmental hospital facilities which in turn damaged the private sector financial turnover [33]. Nevertheless, the generic prescription mandate infuriated groups of the physicians and pharmacists who doubted about the quality, availability of all necessary drugs since the local production did not supply all drugs and to some extent due to the marketing push by the importers [34–36]. But, the market started to bounce back with the shrinkage of the governmental spending after 2 years of the HSEP launch. The HSEP funding shortage resulted in the limitations in insurance coverage and reimbursement to the pharmacies. It did not only influence the retail community pharmacies but also the industry. The important point is that the policymaking has also been compromised in such situations, because the authorities have to deal short term market failures. The industry has not also been willing to invest in new projects, in part because of banking system sanction, hesitation by the international partners and supply chain failure caused by the inefficient supply of the raw materials [1].

## Conclusion

The Iranian pharmaceutical market as a chimeric sector of the economy with a visible hand in play has been growing even during the toughest sanctions. However, it should be noticed that the short-term measures implemented by the HSEP endeavored to alleviate the impacts of the sanctions, but, as the evidence suggests and officials have submitted very recently, the HSEP cannot survive for good. Therefore, sustainable policies need to be introduced to fulfill the missions undertaken by the healthcare system [9, 37]. Nonetheless, the long term consequences of this plan should be evaluated as the evidence keeps evolving and, if needed, adequate corrective measures be applied.

## Data Availability

The datasets used or analyzed during the current study are available from the corresponding author.

## Abbreviations

HSEP: Health sector evolution plan
THE: Total health expenditure
MoH: Ministry of Health and Medical Education
IFDA: Iranian Food and Drug Administration
GDP: Gross domestic product
NHA: National Health Accounts
OOP: Out-of-pocket payment
CAGR: Compound annual growth rate
CR: Concentration ratio
CNS: Central nervous system
ATC: Anatomical therapeutic chemical

## Footnotes

1 Compound annual growth rate
